# PCNT is a prognostic biomarker correlated with tumor immune microenvironment in hepatocellular carcinoma and promotes tumor progression by inhibiting cell cycle arrest

**DOI:** 10.18632/aging.204711

**Published:** 2023-05-19

**Authors:** Xuyang Wang, Jinran Yang, Laibang Luo, Xinchang Li, Youfu Zhang

**Affiliations:** 1 Department of Organ Transplantation, Jiangxi Provincial People’s Hospital, The First Affiliated Hospital of Nanchang Medical College, Nanchang, Jiangxi Province 330006, China

**Keywords:** PCNT, hepatocellular carcinoma, tumor immune microenvironment, prognostic biomarker, cell cycle

## Abstract

Pericentrin (PCNT), a core pericentriolar material protein during mitosis, is involved in tumorigenesis and development in various cancers. However, its role in hepatocellular carcinoma (HCC) remains unclear. Based on public databases and a cohort with 174 HCC patients, we found that PCNT mRNA and protein expression were elevated in HCC tissues and correlated with unfavorable clinicopathological characteristics and prognosis. *In vitro* experiments demonstrated that knockdown PCNT expression inhibited the cell viability, migration, and invasion capacity of HCC cells. Multivariate regression analysis suggested that a high PCNT level was an independent risk factor for poor prognosis. In addition, mutation analysis suggested that PCNT was positively correlated to TMB and MSI but negatively correlated to tumor purity. Moreover, PCNT was significantly negatively correlated with ESTIMATE, immune, and Stromal scores in HCC patients. The PCNT expression level was correlated with immune cell infiltration and immune checkpoint-related gene expression in the tumor microenvironment. The single-cell sequencing analysis suggested that higher PCNT expression level was detected in the malignant cells and immune cells (dendritic cells, monocytes, and macrophages cells) in HCC tissues. Enrichment analysis and functional experiments revealed PCNT promoted tumor progression by inhibiting cell cycle arrest. In conclusion, our studies suggested that PCNT can be a potential prognostic indicator correlated with tumor immune microenvironment, suggesting that PCNT can serve as a novel therapeutic target for HCC.

## INTRODUCTION

Hepatocellular carcinoma (HCC), a kind of malignant with a high degree of heterogeneity, accounts for more than 85% of primary liver cancer [1]. There are more than 90 million new cases and 80 million deaths in 2021 globally, causing an enormous patient health threat and economic burden [2, 3]. With the increasing number of immunotherapeutic and molecular targeted drugs becoming available during the last two decades, almost all patients with HCC could receive systemic therapies [4]. Radical hepatectomy remains the preferred approach for HCC patients with early-stage diseases. However, approximately 70% of patients face recurrence within 5 years after radical surgery due to low tumor purity and high heterogeneity of HCC tissues [5]. There has been a multitude of studies devoted to elucidating the potential mechanism of the initiation and progression of HCC and determining promising prognostic molecular markers [6–8]. The unclarified mechanism hindered therapeutic development. Therefore, searching for novel prognostic biomarkers and therapeutic targets is impending.

Pericentrin (PCNT) is an essential cytoskeleton protein involved in the composition of pericentriolar material, which plays a vital role in maintaining the structure of the mitotic centrosome [9, 10]. Kim et al. demonstrated that the absence of PCNT leads to the failure to recruit pericentriolar material in mitotic centrosomes, thus inhibiting the cell cycle [11]. Aberrantly expression and gene mutation of PCNT generate a series of serious congenital diseases, such as dwarfism, familial intracranial aneurysms., and mental disorders [12–14]. In addition, elevated PCNT expression and mutations usually generate a supernumerary centrosome, which causes the initiation and proliferation of diverse types of cancer cells [15, 16]. Zhou and colleagues demonstrated that a missense SNP rs7279204 in PCNT reduced the protein stability of their host genes and serves as an increased risk of breast cancer [17]. In addition, a prior study has shown that the upregulation expression of PCNT is significantly associated with the recurrence of multiple myeloma [18]. The supernumerary centrosomes and their cancer-promoting role have been validated in HCC [19, 20], but the role of PCNT in HCC has not been investigated.

In the present study, we investigated the PCNT expression level and its prognostic value in HCC through comprehensive bioinformatic analysis and validated by *in vitro* experiments. Our results showed that both PCNT mRNA and protein expression were upregulated in HCC tissues and correlated with unfavorable clinicopathological characteristics and prognosis. In addition, the PCNT expression level was correlated with immune cell infiltration and immune checkpoint-related gene expression in the tumor microenvironment. Finally, the *in vitro* experiments indicate that PCNT promoted tumor progression by inhibiting cell cycle arrest. The main shortcoming of this study is that *in vitro* experiments verified the role of PCNT expression in HCC, but there is lack of *in vivo* experimental verification and requires further experimental research.

## RESULTS

### PCNT mRNA is up-regulated and predicted unfavorable prognosis in HCC

**Figure 1:**
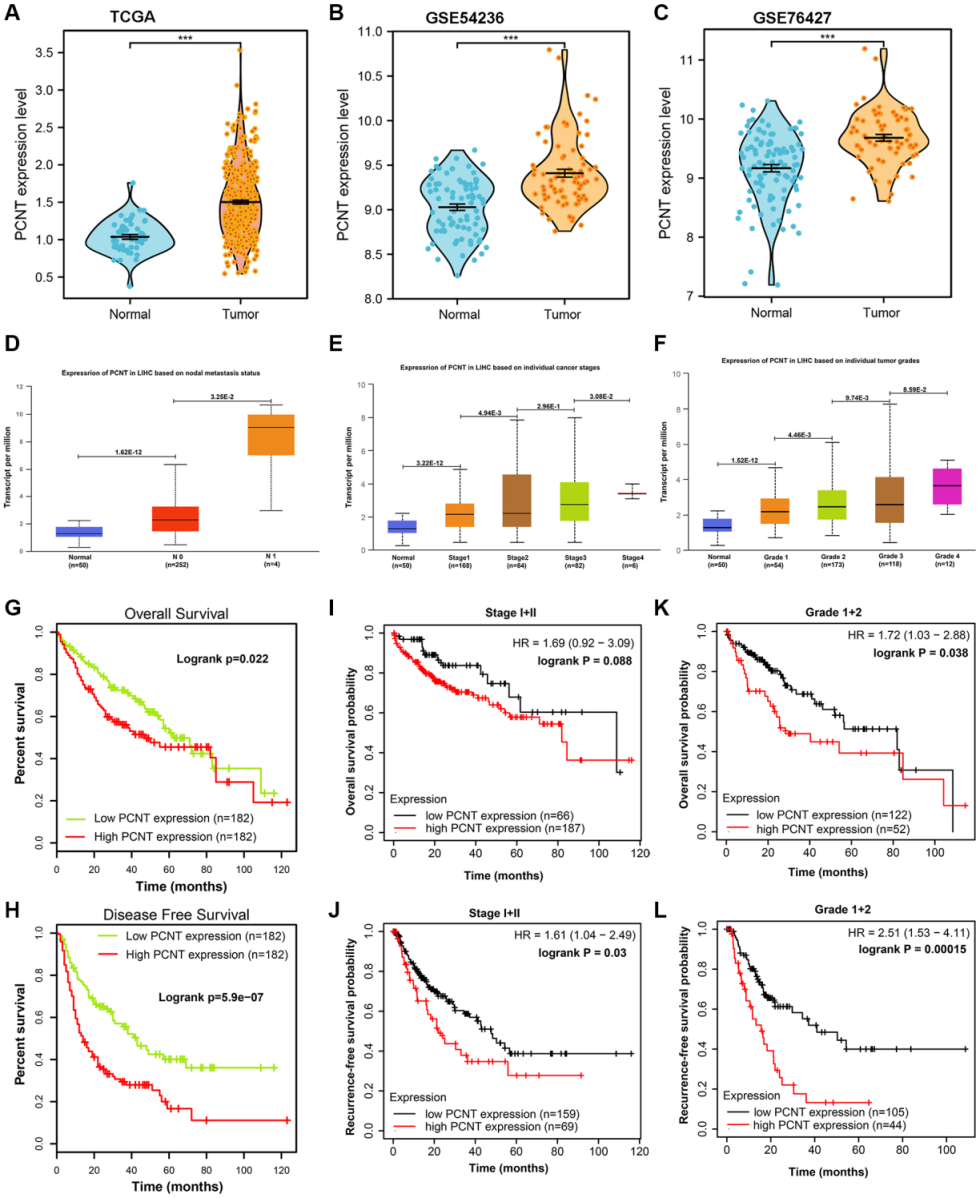
**Expression and clinical role of PCNT mRNA in HCC.** (**A**–**C**) PCNT mRNA levels in HCC and normal tissues in TCGA (**A**), GSE54236 (**B**), and GSE76427 (**C**) datasets. (**D**) PCNT mRNA levels in HCC tissues with lymph node metastasis. (**E**, **F**) PCNT mRNA gradually elevated with the tumor stage (**E**) and grades increased (**F**). (**G**, **H**) Higher PCNT mRNA levels correlated to poor OS (**G**) and disease-free survival (**H**). (**I**, **J**) Higher PCNT mRNA levels correlated to poor OS (**I**) and RFS (**J**) in stage I/II patients. (**K**, **L**) Higher PCNT mRNA levels correlated to poor OS (**K**) and RFS (**L**) in grade I/II patients.

Compared with normal liver tissues, PCNT mRNA was remarkably up-regulated in HCC tissues in TCGA_LIHC, GSE54236, and GSE76427 datasets (Figure 1A–1C). In UALCAN database, the PCNT mRNA was further increased in HCC tissues with lymph node metastasis (Figure 1D). In addition, PCNT mRNA gradually elevated with the tumor stage and grades increased (Figure 1E, 1F). Survival curves indicated that high PCNT mRNA levels predicted unfavorable overall survival (OS) and recurrence-free survival (RFS) periods (Figure 1G, 1H). Noteworthy, high PCNT levels still correlated to poor OS and RFS for patients with early tumor stages (stage I+II, Figure 1I, 1J) and grades (grade I+II, Figure 1K, 1L). The correlation analysis revealed that the high PCNT mRNA level correlated to a higher pathologic stage, pathologic grade, vascular invasion, higher recurrence rate, and low survival rates (Table 1). In addition, the Cox hazard regression analyses suggested that high PCNT mRNA was an independent prognostic factor for OS (*P* = 0.039, HR (95% CI): 1.547 (1.065–3.601)) and RFS (*P* = 0.019, HR (95% CI): 1.616 (1.083–2.412)) of patients with HCC (Table 2).

**Table 1:** Correlation between PCNT expression and clinical characteristics in HCC in the TCGA database (374 cases).

**Characteristics**		***N***	**PCNT mRNA level**		^*^P-Value
			**Low, *n* (%)**	**High, *n* (%)**	
Age (year)	≤60	177	74 (19.8%)	103 (27.6%)	0.004
	>60	196	112 (30%)	84 (22.5%)	
Gender	Female	121	47 (12.6%)	74 (19.8%)	0.004
	Male	253	140 (37.4%)	113 (30.2%)	
T stage	T1/T2	278	152 (41%)	126 (33.9%)	**<0.001**
	T3/T4	93	32 (8.7%)	61 (16.4%)	
N stage	N0	254	123 (47.7%)	131 (50.8%)	0.124
	N1	4	0 (0%)	4 (1.6%)	
M stage	M0	268	134 (49.3%)	134 (49.3%)	0.122
	M1	4	4 (1.5%)	0 (0%)	
Pathologic stage	I/II	100	155 (41.5%)	115 (32.9%)	**<0.001**
	III/IV	50	30 (8.5%)	60 (17.1%)	
Serum AFP level	≤400	215	123 (43.9%)	92 (32.9%)	0.063
	>400	65	28 (10%)	37 (13.2%)	
Histologic grade	G1/G2	233	129 (35%)	104 (28.2%)	**0.039**
	G3/G4	136	55 (14.9%)	81 (21.9%)	
Child-Pugh class	A	219	125 (51.9%)	94 (39%)	0.358
	B/C	22	10 (4.1%)	12 (5%)	
Vascular invasion	No	208	113 (35.5%)	95 (29.9%)	**0.005**
	Yes	110	56 (17.6%)	54 (17%)	
Recurrence	No	202	116 (32.7%)	86 (24.2%)	**0.007**
	Yes	153	65 (18.3%)	88 (24.8%)	
Survival status	Alive	191	107 (28.6%)	84 (22.5%)	**0.023**
	Dead	183	80 (21.4%)	103 (27.5%)	

Abbreviations: T: tumor; N: node; M: metastasis; AFP: alpha fetoprotein. ^*^P-Value < 0.05 were considered statistically significant.

**Table 2:** Univariate and multivariate cox regression analysis of overall survival and recurrence-free survival in patients with hepatocellular carcinoma.

**Variables**		**Overall survival**	^*^P-Value	**Recurrence-free survival**	^*^P-Value
		**HR (95% CI)**		**aHR (95% CI)**	
**Univariate analyses**					
Age (year)	≤60 vs. >60	1.205 (0.850–1.708)	0.295	0.960 (0.718–1.284)	0.783
Gender	Male vs. Female	0.793 (0.557–1.130)	0.2	0.982 (0.721–1.338)	0.909
T stage	T1/T2 vs. T3/T4	2.598 (1.826–3.697)	**<0.001**	2.177 (1.590–2.980)	**<0.001**
N stage	N0 vs. N1	2.029 (0.497–8.281)	0.324	1.370 (0.338–5.552)	0.659
M stage	M0 vs. M1	4.077 (1.281–12.973)	**0.017**	3.476 (1.091–11.076)	**0.035**
Pathologic stage	I/II vs. III/IV	2.504 (1.727–3.631)	**<0.001**	1.152 (0.853–1.557)	**<0.001**
Serum AFP level	≤400 vs. >400	1.075 (0.658–1.759)	0.772	1.045 (0.698–1.563)	0.832
Histologic grade	G1/G2 vs. G3/G4	1.091 (0.761–1.564)	0.636	1.152 (0.853–1.557)	0.355
Child-Pugh class	A vs. B/C	1.643 (0.811–3.330)	0.168	1.395 (0.765–2.545)	0.277
Vascular invasion	Yes vs. No	1.344 (0.887–2.035)	0.163	1.676 (1.196–2.348)	**0.003**
PCNT mRNA level	High vs. Low	1.869 (1.186–2.947)	**0.007**	1.706 (1.271–2.289)	**<0.001**
**Multivariate analyses**					
T stage	T1/T2 vs. T3/T4	1.550 (0.210–11.425)	0.667	0.342 (0.075–1.559)	0.166
M stage	M0 vs. M1	2.031 (0.599–6.886)	0.255	3.028 (0.827–11.092)	0.094
Pathologic stage	I/II vs. III/IV	1.821 (1.245–13.534)	**0.048**	4.266 (1.929–19.589)	**0.042**
Vascular invasion	Yes vs. No	1.277 (0.907–2.306)	0.148	1.388 (0.924–2.085)	0.114
PCNT mRNA level	High vs. Low	1.547 (1.065–3.601)	**0.039**	1.616 (1.083–2.412)	**0.019**

Abbreviations: HR: Hazard ratio; aHR: Adjusted hazard ratio; CI: confidential interval; PCNT: Pericentrin; T: tumor; N: node; M: metastasis; AFP: alpha fetoprotein. ^*^P-Value < 0.05 were considered statistically significant.

### PCNT protein is up-regulated and predicted unfavorable prognosis in a cohort with 174 HCC patients

**Figure 2:**
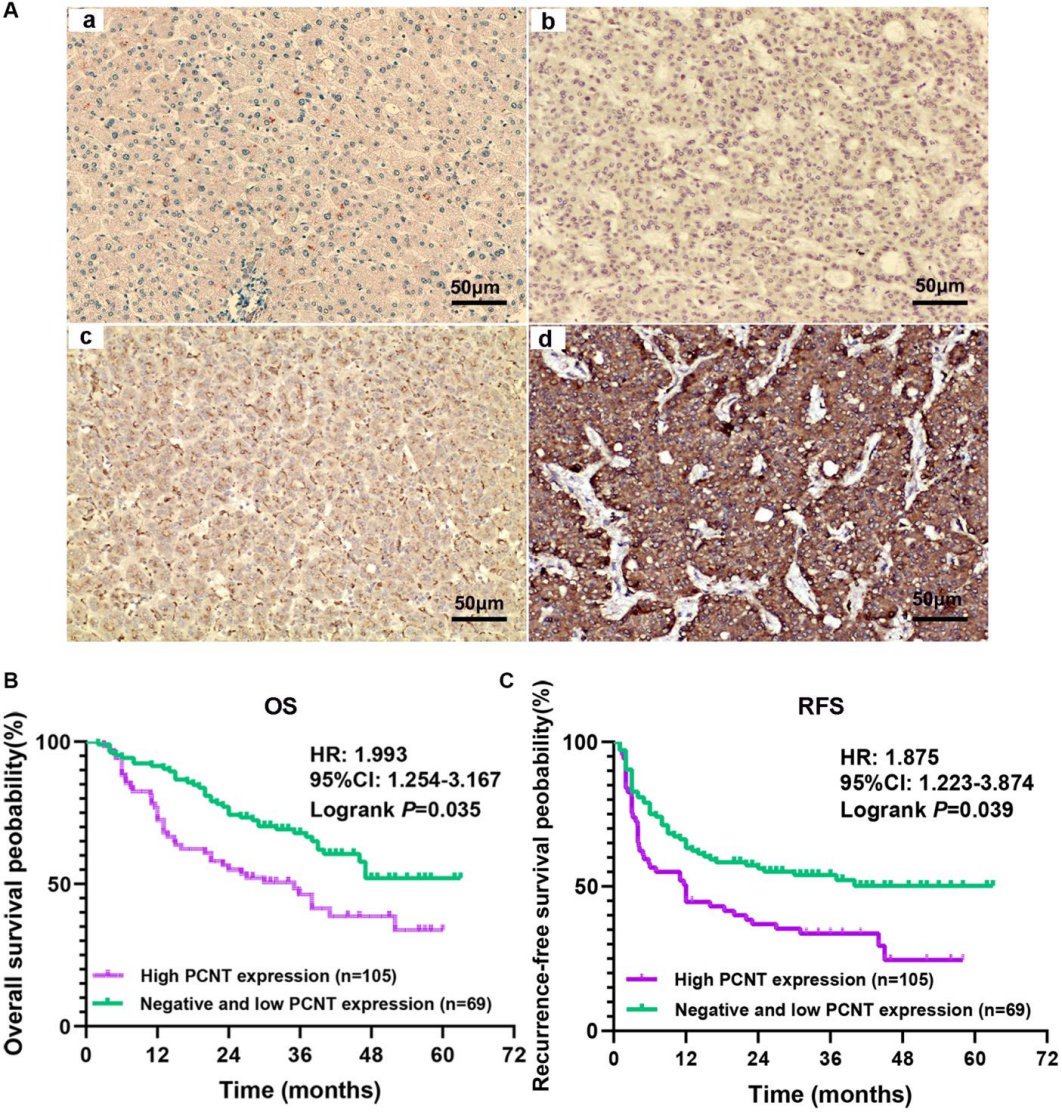
**Expression and prognostic role of PCNT protein in HCC.** (**A**) The representative images of adjacent normal liver tissues (**a**) and negative (**b**), low (**c**), and high (**d**) PCNT protein expression HCC tissues. (**B**, **C**) Higher PCNT protein expression correlated to shorter OS (**B**) and RFS (**C**) periods in HCC patients.

We further investigated the PCNT protein level in HCC tissues and its prognostic value in a cohort with 174 HCC patients. PCNT protein level was remarkably elevated in the vast majority of HCC tissues compared with adjacent normal liver tissues. Based on the IHC staining, negative, low, and high PCNT protein expression among 174 HCC patients was 47, 58, and 69, respectively. We presented representative pictures of PCNT protein expression in adjacent normal liver tissues and HCC tissues (Figure 2A). Correlation analysis indicated that high PCNT protein expression correlated to a higher TNM stage, poorly-tumor differentiation, vascular invasion, higher recurrence rate, and low survival rates (Table 3). In addition, consistent with the prognostic role of PCNT mRNA, multivariate regression analysis suggested that high PCNT protein level was an independent risk factor for OS (*P* = 0.025, HR (95% CI): 1.201 (1.024–1.409)) and RFS (*P* = 0.016, HR (95% CI): 1.192 (1.033–1.376)) of HCC patients (Table 4). Survival curves validated that high PCNT protein expression correlated to shorter OS and RFS periods (Figure 2B, 2C).

**Table 3:** Correlation between PCNT protein expression and clinicopathologic features in 174 patients with hepatocellular carcinoma.

**Characteristics**		***N***	**PCNT protein level**		**χ²**	^*^P-Value
			**High (*n*)**	**Low (*n*)**		
Age (year)	>55	112	47	65	0.700	0.403
	≤55	62	22	40		
Gender	Male	144	51	93	6.270	0.012
	Female	30	18	12		
Tumor size (cm)	>5 cm	94	42	52	2.158	0.142
	≤5 cm	80	27	53		
TNM stage	I/II	97	26	71	15.128	**<0.001**
	III	77	43	34		
Serum AFP level	>400 ng/ml	74	29	45	0.012	0.974
	≤400 ng/ml	100	40	60		
Tumor location	Left	57	24	33	0.213	0.645
	Right	117	45	72		
Tumor differentiation	Poorly	21	14	8	6.360	**0.042**
	Moderately	112	42	70		
	Well	41	13	27		
HBsAg	Positive	85	36	49	0.505	0.477
	Negative	89	33	56		
Edmonson grade	I	34	17	17	1.890	0.169
	II–IV	140	52	88		
Child-Pugh class	A	85	36	49	0.505	0.477
	B	89	33	56		
Vascular invasion	Yes	82	39	43	4.051	**0.044**
	No	92	30	62		
Tumor encapsulation	Yes	111	41	70	0.947	0.331
	No	63	28	35		
Recurrence	Yes	96	47	49	7.745	**0.005**
	No	78	22	56		
Survival status	Alive	93	29	64	5.993	**0.014**
	Dead	81	40	41		

Abbreviations: PCNT: Pericentrin; TNM: tumor node metastasis; AFP: alpha fetoprotein. ^*^P-Value < 0.05 were considered statistically significant. Bold value considered statistically significant.

**Table 4:** Univariate cox regression analysis of overall survival and recurrence-free survival in 174 patients with hepatocellular carcinoma.

**Variables**		**Overall survival**	^*^P-Value	**Recurrence-free survival**	^*^P-Value
		**HR (95% CI)**		**aHR (95% CI)**	
**Univariate**					
Age (year)	>55 vs. ≤55	0.844 (0.533–1.303)	0.425	0.714 (0.474–1.073)	0.105
Gender	Male vs. Female	1.675 (0.991–2.831)	0.054	1.728 (1.073–2.785)	**0.025**
Tumor size (cm)	>5 vs. ≤5	1.616 (1.220–2.414)	**<0.001**	1.484 (1.139–1.935)	**0.003**
TNM stage	I/II vs. III	2.778 (1.944–3.971)	**<0.001**	1.775 (1.270–2.483)	**<0.001**
Serum AFP level	>400 vs. ≤400	2.021 (1.303–3.134)	**0.002**	1.724 (1.154–2.574)	**0.008**
Tumor location	Left vs. Right	0.803 (0.510–1.266)	0.345	1.159 (0.749–1.791)	0.508
Tumor differentiation	Well vs. Moderately/Poorly	1.352 (0.771–2.371)	0.292	1.462 (0.875–2.443)	0.147
HBsAg	Positive vs. Negative	1.504 (0.969–2.332)	0.065	1.140 (0.764–1.701)	0.522
Edmonson grade	I vs. II–IV	0.925 (0.520–1.646)	0.79	0.792 (0.463–1.356)	0.396
Child-Pugh class	A vs. B	3.973 (2.459–6.420)	**<0.001**	1.738 (1.159–2.607)	**0.008**
Vascular invasion	Yes vs. No	1.637 (1.054–2.542)	**0.028**	2.208 (1.466–3.327)	**<0.001**
Tumor encapsulation	Yes vs. No	0.637 (0.410–0.988)	**0.044**	0.249 (0.164–0.377)	**<0.001**
PCNT protein level	High vs. Low	1.337 (1.134–1.575)	**<0.001**	1.288 (1.108–1.496)	**<0.001**
**Multivariate**					
Gender	Male vs. Female			1.170 (0.688–1.989)	0.562
Tumor size (cm)	>5 vs. ≤5	0.877 (0.563–1.365)	0.56	1.082 (0.763–1.533)	0.659
TNM stage	I/II vs. III	2.001 (1.275–3.141)	**0.003**	1.107 (0.756–1.620)	0.602
Serum AFP level	>400 vs. ≤400	1.643 (1.045–2.584)	**0.031**	1.661 (1.106–2.496)	**0.014**
Child-Pugh class	A vs. B	3.001 (1.827–4.928)	**<0.001**	1.417 (0.927–2.166)	0.107
Vascular invasion	Yes vs. No	1.296 (0.813–2.067)	0.276	2.180 (1.427–3.330)	**<0.001**
Tumor encapsulation	Yes vs. No	0.869 (0.540–1.333)	0.476	0.260 (0.167–0.405)	**<0.001**
PCNT protein level	High vs. Low	1.201 (1.024–1.409)	**0.025**	1.192 (1.033–1.376)	**0.016**

Abbreviations: aHR: adjusted Hazard ratio; CI: confidential interval; TNM: tumor node metastasis; AFP: alpha fetoprotein; PCNT: Pericentrin. ^*^P-Value < 0.05 were considered statistically significant. Bold value considered statistically significant.

### Building and validating a predictive nomogram

**Figure 3:**
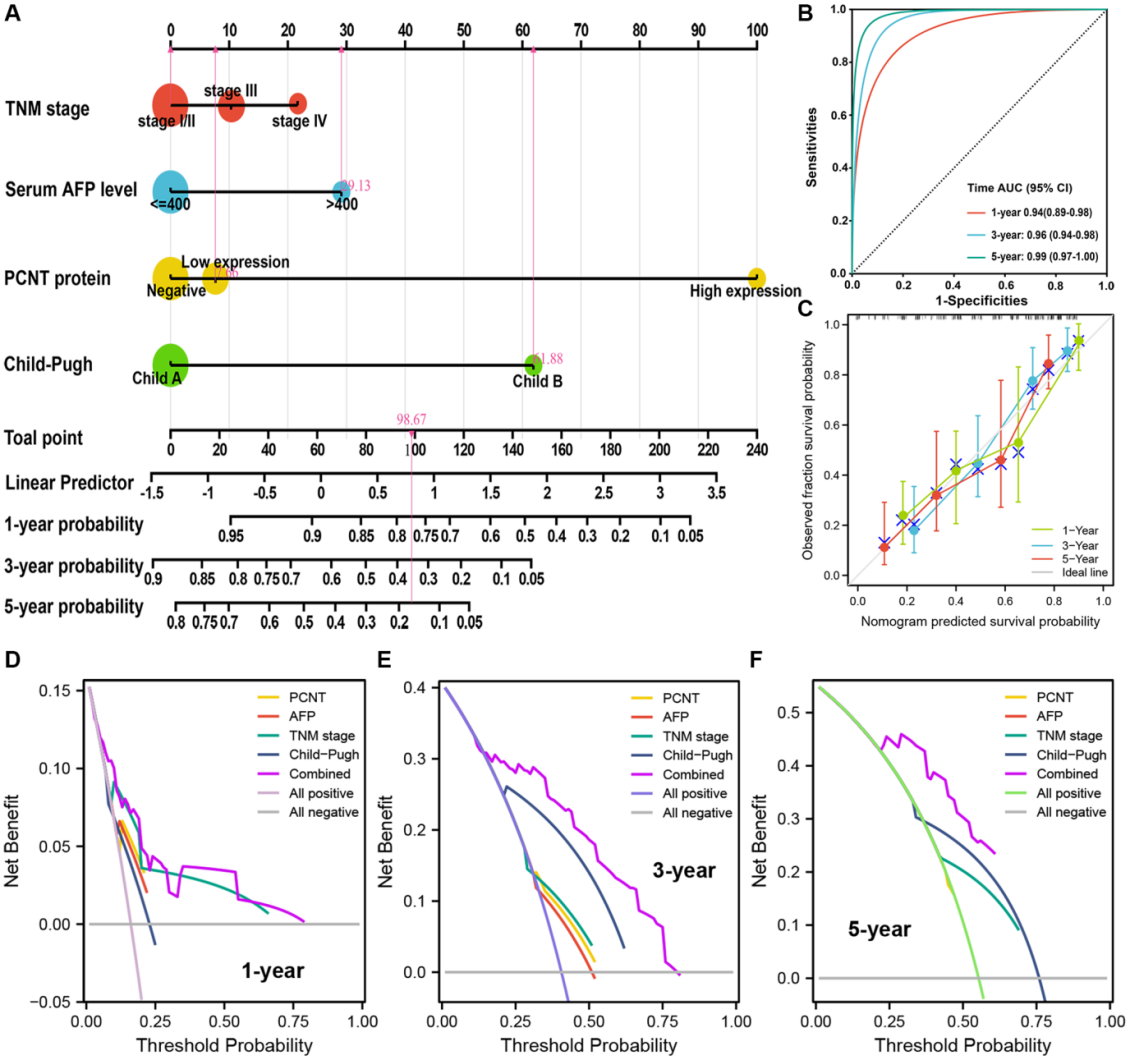
**Nomogram analyses of the selected prognostic factors.** (**A**) A nomogram was built using four independent prognostic factors. (**B**) Time-dependent ROC was employed to weigh up the performance of nomogram model. (**C**) Calibration curves for nomogram model related to 1-, 3-, and 5-year OS. (**D**–**F**) DCA for nomogram model related to 1-, 3-, and 5-year OS.

Based on the multivariate regression analysis of 174 HCC patients, TNM stage, serum AFP level, PCNT protein level, and Child-Pugh class were independent risk factors for OS. These factors were incorporated to build a nomogram for accurate prediction of 1-, 3-, and 5-year OS rates (Figure 3A). The C-index of the nomogram was 0.774 (*p* = 1.495e-24, 95% CI (0.721–0.829)). The AUC under time-dependent ROC of 1-, 3-, and 5-year OS were 0.94, 0.96, and 0.99, respectively, suggesting an excellent prediction performance of the nomogram (Figure 3B). The calibration curves also validated the satisfactory prognostic effect of the nomogram (Figure 3C). Besides, as shown in the DCA curves, the combined nomogram model exhibited the highest net benefits both for 1-, 3-, and 5-year OS prediction compared with the signal risk factor (Figure 3D–3F).

### PCNT promoted cell proliferation and invasion in HCC

**Figure 4:**
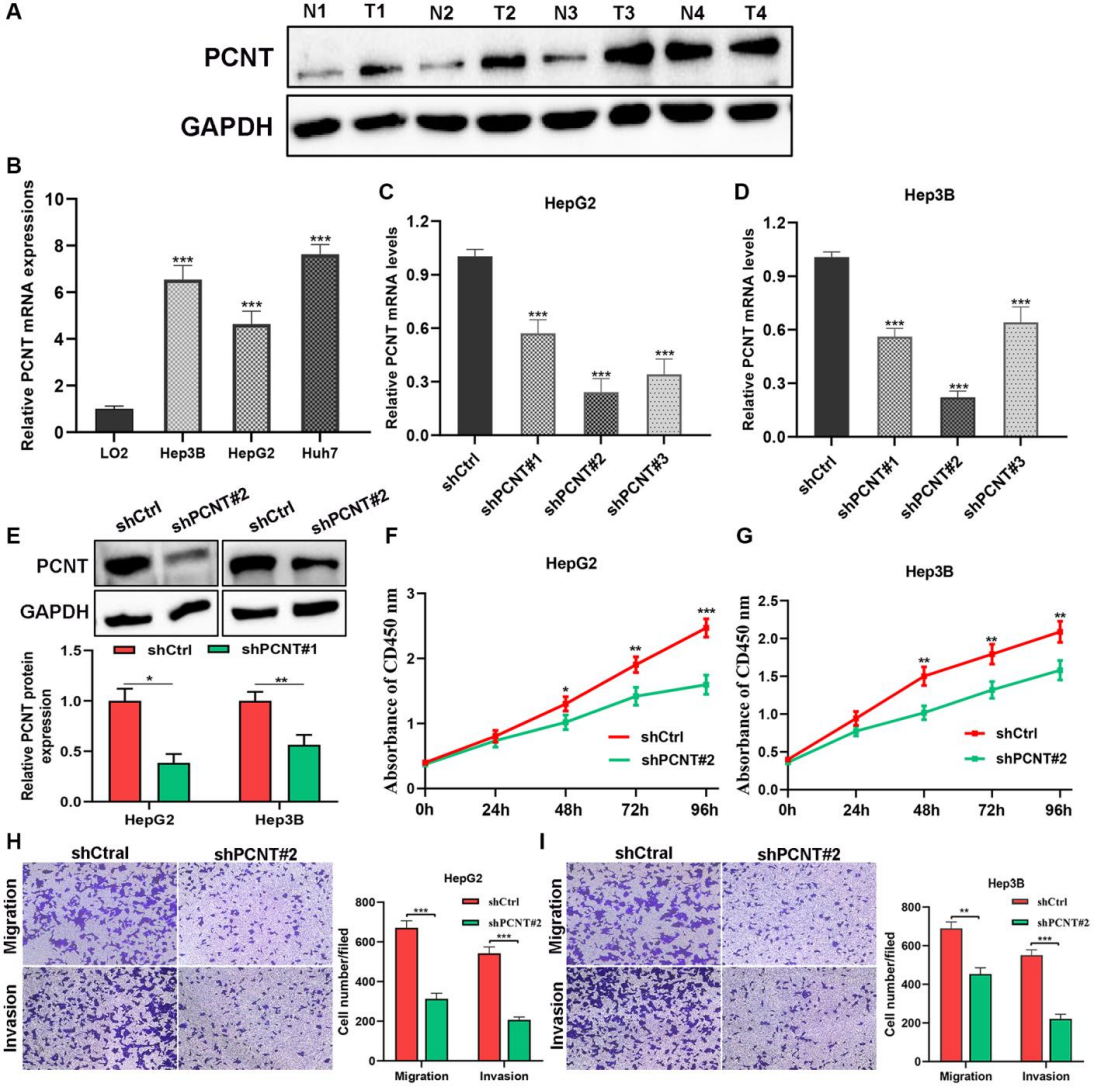
***In vitro* experiments validated PCNT expression and pro-oncogenic effects.** (**A**) PCNT protein level in 4 paired HCC tissues and their adjacent normal liver tissues. (**B**) PCNT mRNA levels in LO2, Hep3B, HepG2, and Huh7 cell lines. (**C**, **D**) PCNT mRNA levels were down-regulated by shPCNT transfection in HepG2 (**C**) and Hep3B (**D**) cell lines. (**E**) Western blot assay validated the knockdown effects of shPCNT#2. (**F**, **G**) CCK-8 assays detected the cell viability of HepG2 (**F**) and Hep3B (**G**) cells. (**H**, **I**) The migration and invasion capacities were examined by transwell assays in HepG2 (**H**) and Hep3B (**I**) cell lines. ^*^P < 0.05, ^**^P < 0.01, ^***^P < 0.001.

The western blot was performed and found PCNT protein level was remarkably increased in 4 HCC samples compared with paired adjacent noncancerous samples (Figure 4A). We then performed cellular experiments to investigate the role of PCNT in the biological behaviors of HCC cells. The RT-qPCR validated that increase of PCNT mRNA level was detected in Hep3B, HepG2, and Huh7 cell lines (Figure 4B). After being transfected with shPCNT, HepG2 and Hep3B exhibited a markedly decreased PCNT mRNA (Figure 4C, 4D). We selected shPCNT#2 to conduct subsequent experiments for its best knockdown effects. The knockdown efficiency of shPCNT#2 in HepG2 and Hep3B cell lines was further validated using the western blot assays (Figure 4E). The CCK-8 assays demonstrated that knockdown PCNT expression in HepG2 and Hep3B cells led to decreases in cell viability (Figure 4F, 4G). Besides, we conducted transwell assays and found the migration and invasion capacities of HCC cells were markedly decreased after PCNT knockdown (Figure 4H, 4I).

### PCNT mRNA level correlated to somatic mutations, TMB, and MSI in HCC patients

**Figure 5:**
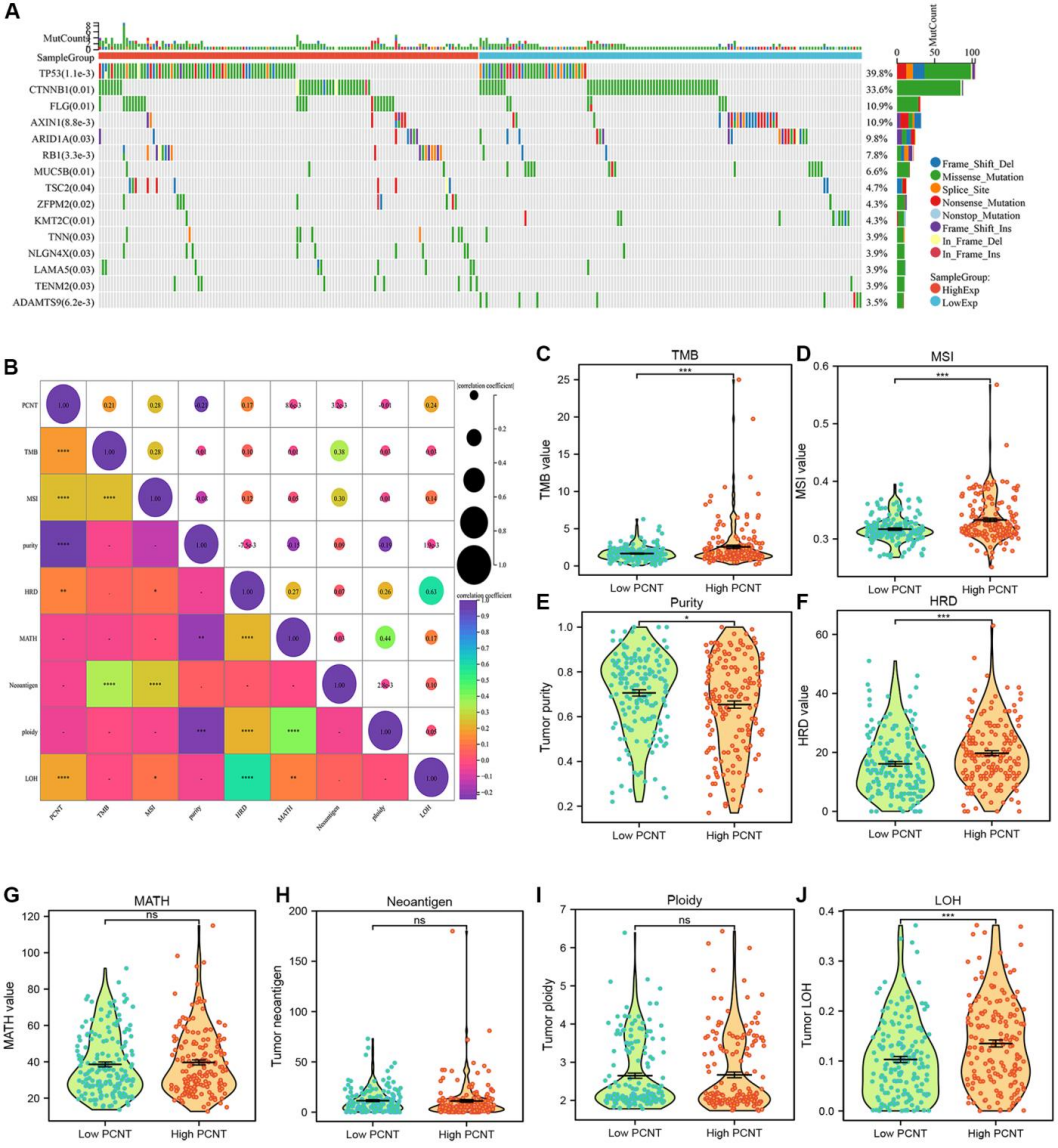
**Associations between PCNT expression level with mutation profile.** (**A**) The waterfall plot presented the mutation difference of top 15 genes between high and low PCNT mRNA groups. (**B**) PCNT mRNA level positively correlated with TMB, MSI, HRD, and LOH, but negatively correlated with tumor purity. (**C**–**J**) The difference of TMB (**C**), MSI (**D**), tumor purity (**E**), HRD (**F**), MATH (**G**), tumor neoantigen (**H**), tumor ploidy (**I**), and LOH (**J**) between high and low PCNT expression groups.

Somatic mutation data downloaded from the TCGA database were employed to investigate the correlations between PCNT mRNA level and mutation profiles. The top 15 genes mutation was found in 255 (69.1%) of 369 HCC patients. As shown in the waterfall plot, TP53, CTNNB1, FLG, AXIN1, and ARID1A presented a significant mutation difference between high and low PCNT mRNA groups (Figure 5A). Then, Pearson’s correlation analysis was employed to investigate the associations of PCNT expression with TMB, MSI, MATH, tumor purity, tumor neoantigen, tumor ploidy, HDR, and LOH. As shown in Figure 5B, PCNT mRNA level was markedly positively correlated to TMB, MSI, HRD, and LOH, whereas negatively associated with tumor purity (Figure 5B). Besides, the Mann–Whitney rank sum test validated that patients with higher PCNT expression have higher TMB, MSI, and HRD values but lower tumor purity (Figure 5C–5J).

### PCNT was involved in tumor immune microenvironment in HCC

**Figure 6:**
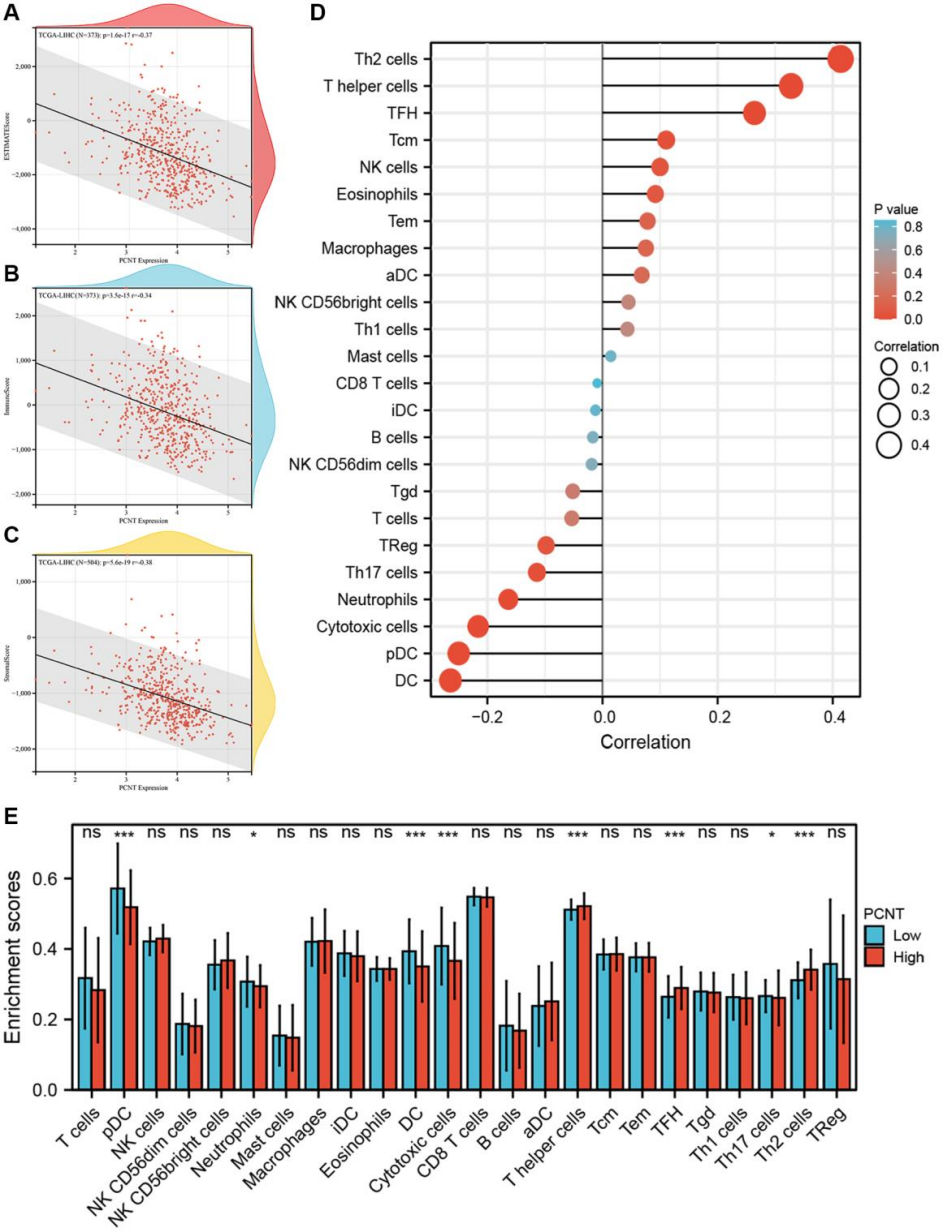
**Associations between PCNT expression level with immune cell infiltration.** (**A**–**C**) PCNT mRNA levels negatively correlated with Estimate score (**A**), Immune score (**B**), and Stromal score (**C**). (**D**) Pearson’s correlations analysis between PCNT mRNA levels with 24 immune cell infiltration. (**E**) The differential abundance of 24 immune cells between high and low PCNT mRNA levels groups.

**Figure 7:**
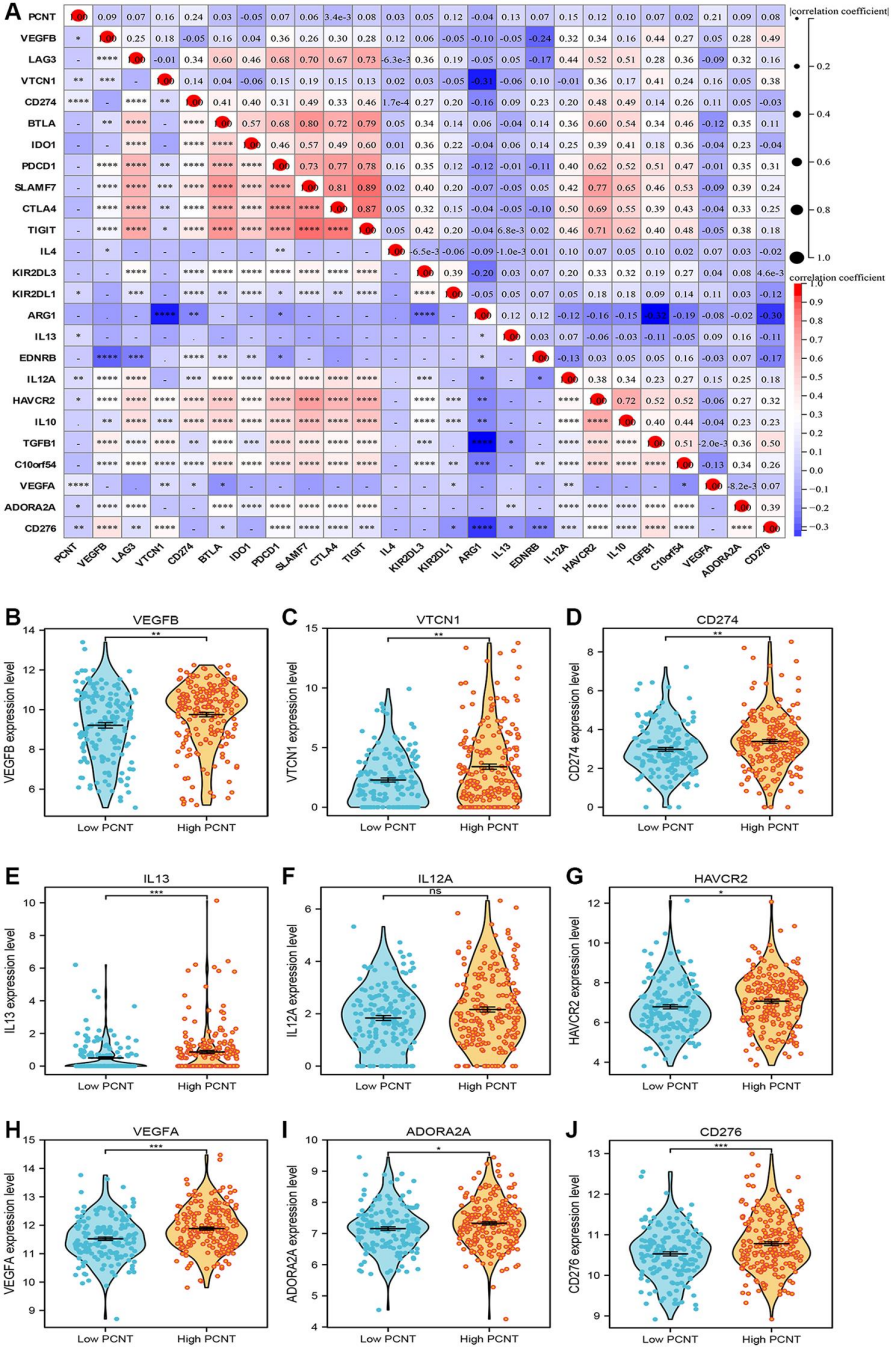
(**A**) The associations between PCNT and immune checkpoint inhibitor-related gene expression levels. (**B**–**J**) HCC tissues with higher PCNT expression have a higher level of VEGFB (**B**), VTCN1 (**C**), CD274 (**D**), IL13 (**E**), IL12A (**F**) HAVCR2 (**G**), VEGFA (**H**), ADORA2A (**I**), and CD276 (**J**) expression. ^*^P < 0.05, ^**^P < 0.01, ^***^P < 0.001, ^****^P < 0.0001.

The immune cell infiltration and expression of immune checkpoint inhibitor-related genes in the tumor microenvironment play key roles in the initiation, development, and immunotherapy resistance of HCC [21–23]. Therefore, we investigated the relationship between PCNT expression with tumor immune microenvironment. Pearson’s correlation analysis demonstrated that PCNT was associated negatively with the ESTIMATE, immune, and stromal scores in HCC (Figure 6A–6C). In addition, the ssGSEA algorithm with Spearman’s analysis was employed to investigate relationships between PCNT expression with 24 immune cells infiltrating level and found that PCNT was associated positively with Th2 cells, T helper cells, and Follicular helper T cells, but negatively correlated to dendritic cells, pDC cells, and cytotoxic cells (Figure 6D). Moreover, the CIBERSORT algorithm validated that HCC tissues with higher PCNT expression have a higher proportion of T helper cells, TFH cells, and Th2 cells, but a lower proportion of dendritic cells, pDC cells, and cytotoxic cells (Figure 6E). The associations between PCNT and immune checkpoint inhibitor-related gene expression levels were investigated and exhibited significantly positive correlations (Figure 7A). Then, the Mann–Whitney rank sum test further validated that HCC tissues with higher PCNT expression have a higher level of VEGFB, VTCN1, CD274, IL13, HAVCR2, VEGFA, ADORA2A, and CD276 expression (Figure 7B–7J).

### PCNT expression at the single-cell level

**Figure 8:**
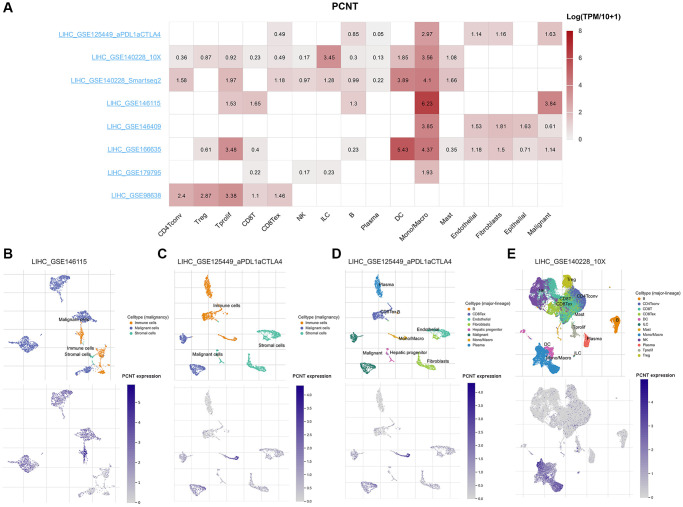
**PCNT expression at the single-cell level.** (**A**) The matrix heat map presented the PCNT mRNA levels in malignant, stromal, and different types of cells. (**B**) The PCNT mRNA levels and cell distributions in LIHC_GSE146115 (**B**) and LIHC_GSE125449a_PDL1aCTLA4 (**C**) datasets. (**D**) Normal hepatic progenitors detected low PCNT mRNA levels in LIHC_GSE125449a_PDL1aCTLA4 dataset. (**E**) The distributions and PCNT mRNA levels in different types of immune cells in LIHC_GSE140228_10X dataset.

We next investigated the PCNT expression at the single-cell levels. The matrix heat map presented the PCNT mRNA levels in malignant, stromal, and different types of cells (Figure 8A). In the LIHC_GSE146115 and LIHC_GSE125449a_PDL1aCTLA4 datasets, PCNT was mainly detected in malignant and immune cells (Figure 8B, 8C). In addition, the LIHC_GSE125449a_PDL1aCTLA4 dataset exhibited that normal hepatic progenitors have low levels of PCNT mRNA compared with malignant and immune cells (Figure 8D). In the LIHC_GSE140228_10X dataset, PCNT was mainly detected in the monocytes, macrocytes, and dendritic cells (Figure 8E). These results revealed that PCNT may function in immune cells and malignant cells.

### Prognosis and GO functional analysis of PCNT co-expressed genes

**Figure 9:**
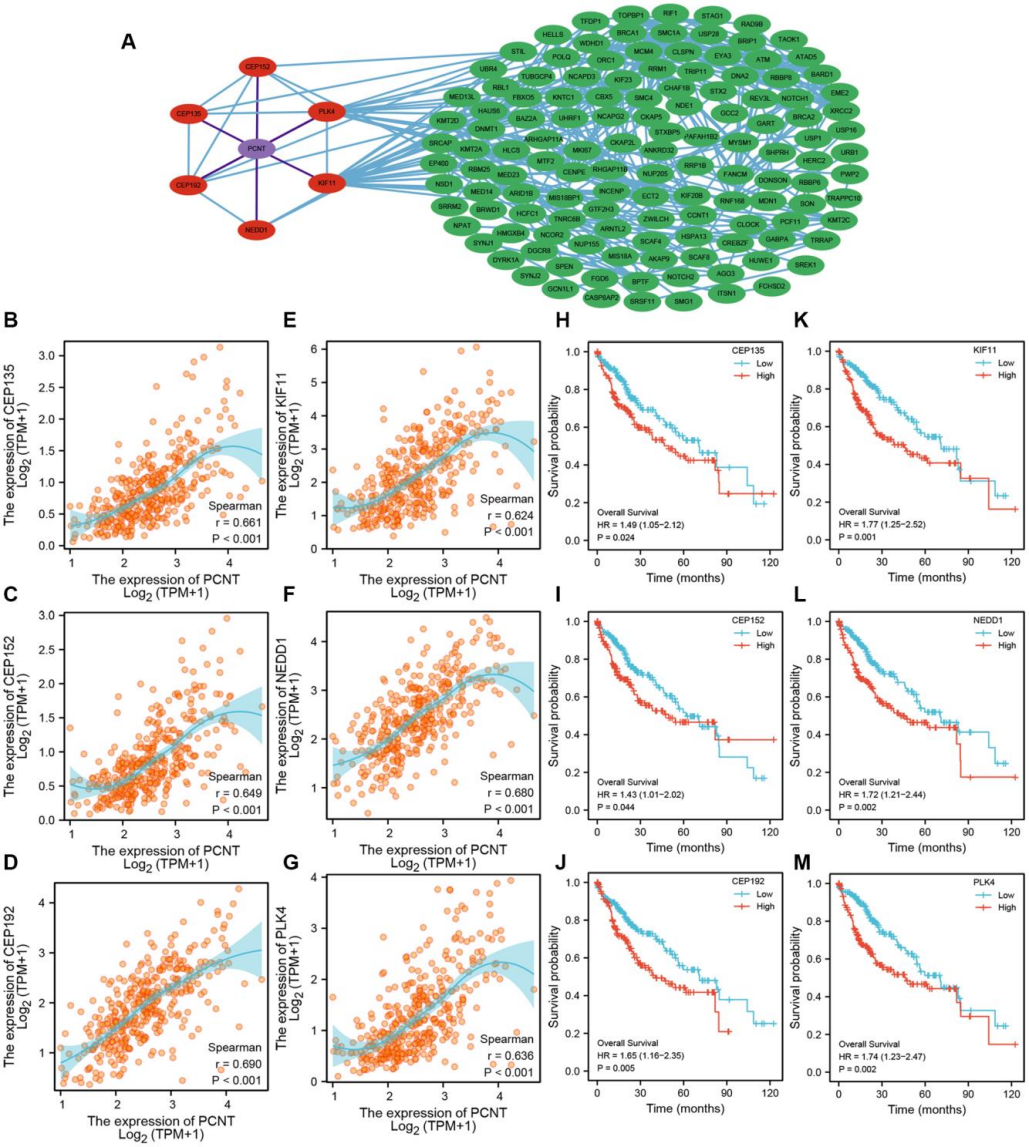
**PPI network and prognostic value of PCNT co-expressed genes.** (**A**) PPI network exhibited six proteins directly interacted with PCNT. (**B**–**G**) PCNT expression was associated positively with CEP135 (**B**), CEP152 (**C**), CEP192 (**D**), PLK4 (**E**), KIF11 (**F**), and NEDD1 (**G**) in HCC. (**H**–**M**) Patients with higher expression of CEP135 (**H**), CEP152 (**I**), CEP192 (**J**), PLK4 (**K**), KIF11 (**L**), and NEDD1 (**M**) have shorter OS periods in HCC.

**Figure 10:**
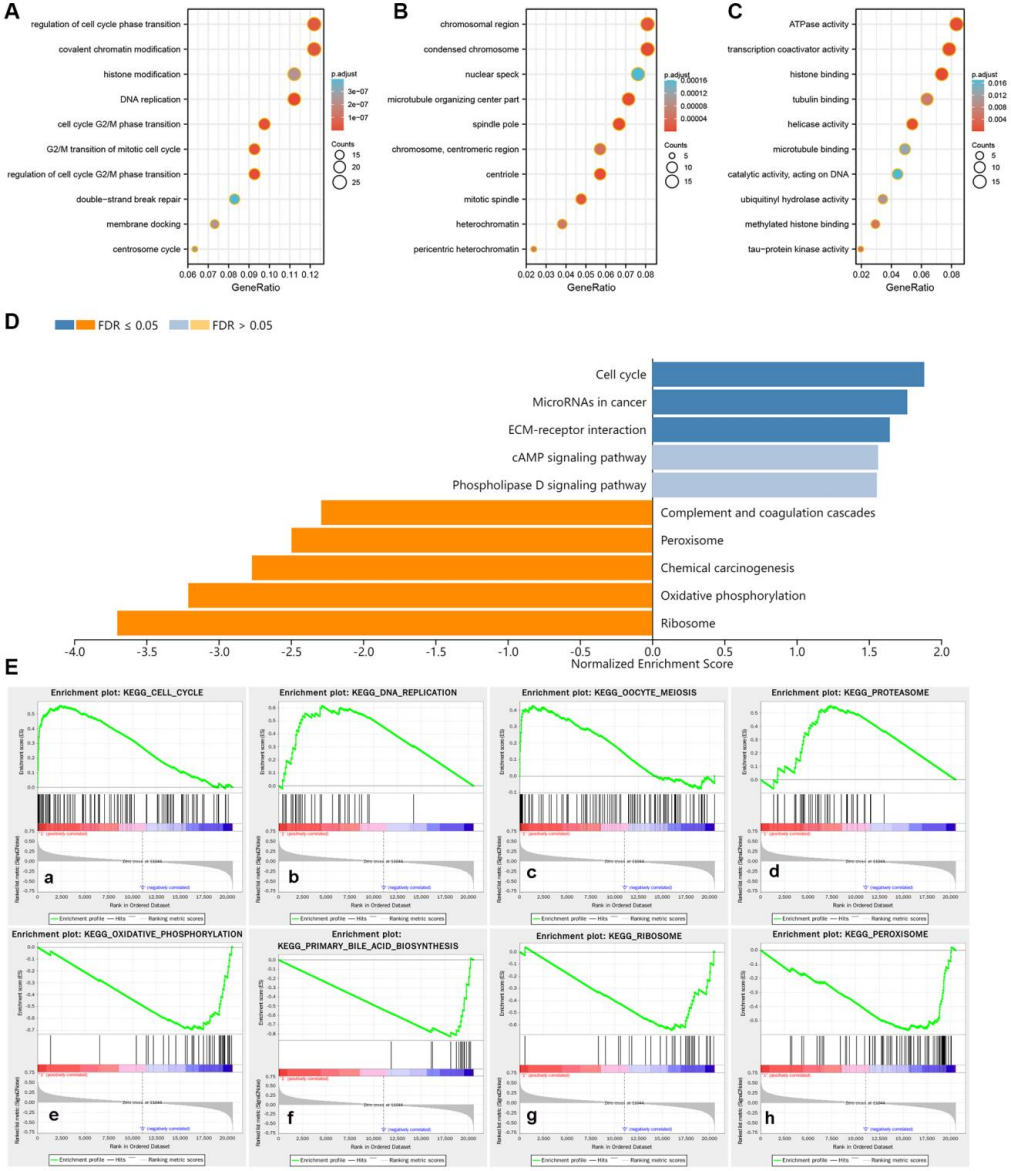
**Potential biological functions and enriched pathways of PCNT.** (**A**–**C**) The terms of Biological process (**A**), Cellular component (**B**), and Molecular function (**C**) in GO analysis. (**D**) Enrichment analysis of KEGG terms for PCNT involved. (**E**) Gene sets were markedly positively and negatively enriched in higher PCNT expression patients.

A total of 139 co-expressed genes of PCNT were identified in the LinkedOmics databases. As shown in Figure 9A, we constructed a PPI network to visualize interactions between these genes with PCNT (Figure 9A). Six genes (CEP135, CEP152, CEP192, PLK4, KIF11, and NEDD1) directly interacted with PCNT. Pearson’s analysis validated these genes were associated positively with PCNT in TCGA database (Figure 9B–9G). In addition, survival curves indicated that higher expression of these genes correlated to shorter OS periods in patients with HCC (Figure 9H–9M). By extension, it is reasonable to speculate that PCNT plays protumorigenic role by interacting with these co-expression genes. We next performed GO functional analysis on 139 co-expressed genes to gain insight into the biological function of PCNT. We presented the top 10 significant terms of Biological process, Molecular function, and Cell component. Results demonstrated that PCNT co-expressed genes were mainly related to the regulation of cell cycle phase transition, chromosomal region, and ATPase activity (Figure 10A–10C).

### PCNT promoted tumor progression by inhibiting cell cycle arrest

**Figure 11:**
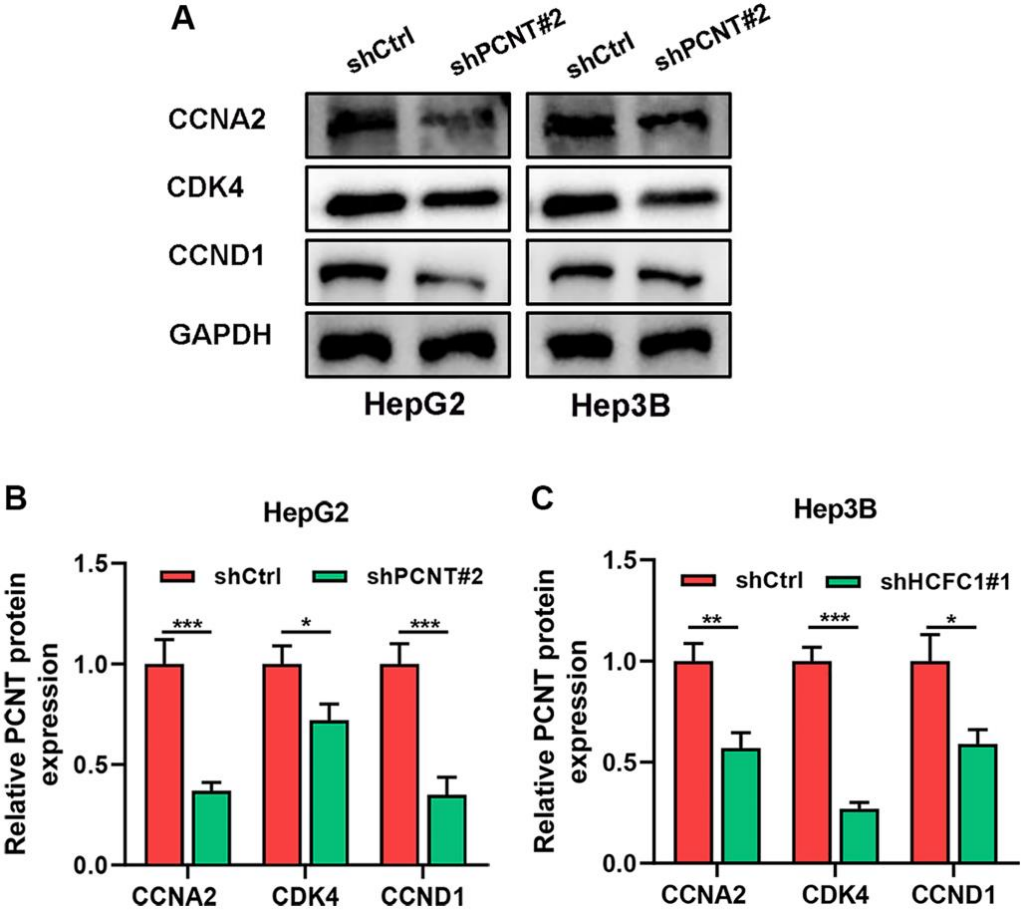
**PCNT Knockdown promoted the cell cycle arrest in HCC cells.** (**A**–**C**) Western blots images (**A**) and statistical analysis of CCNA2, CDK4, and CCND1 expression in HepG2 (**B**) and Hep3B (**C**) cell lines. ^*^P < 0.05, ^**^P < 0.01, ^***^P < 0.001.

We performed KEGG pathways in the LinkedOmics database and uncovered that PCNT was involved in Cell cycle, ECM-receptor interaction, and other cancer-related signaling (Figure 10D). In addition, GSEA analysis validated that gene sets of the cell cycle and other cancer-related pathways were markedly positively enriched in higher PCNT expression patients (Figure 10E). *In vitro* experiments were performed to validate this bioinformatics-based finding. Western blot assays uncovered that protein levels of three cell cycle-related proteins (CCNA2, CDK4, and CCND1) significantly decreased in HepG2 and Hep3B cell lines after knockdown PCNT expression (Figure 11A–11C), suggesting that PCNT might affect the progression of HCC through regulating the cell cycle signal.

## DISCUSSION

HCC is one of the most common malignancies with a high degree of heterogeneity in the digestive system and was the second leading cause of cancer-related death globally [24, 25]. Increased gradual morbidity and mortality exhort many scholars to identify novel prognostic markers and therapeutic targets for efficacious treatments [26].

PCNT is an essential cytoskeleton protein involved in the composition of the mitotic centrosome [27]. Centrosome replication occurs during the mitotic phase and occurs once in each cell cycle. Aberrant expression of PCNT results in numerical or structural abnormalities of centrosome, thereby triggering a perturbed cell-cycle [19, 28]. PCNT aberrant expression and mutations usually generate the supernumerary centrosome, thereby leading to the initiation and proliferation of diverse types of cancer cell [15, 16]. PCNT was up-regulated in tumor endothelium compared with adjacent normal glands in prostate cancer patients and drives tumor growth and angiogenesis [29]. In addition, bladder cancer, B-acute lymphoblastic leukemia, breast cancer, and melanoma were also detected with upregulated PCNT levels and their pro-oncogenic roles [30–33]. However, the role of PCNT in HCC has not been investigated.

Herein, we analyzed the PCNT expression and clinical prognostic significance based on multiple public databases and an HCC cohort. We are pleased to note that PCNT was remarkably overexpressed in HCC tissues at mRNA level in TCGA_LIHC, GSE54236, and GSE76427 datasets. IHC and western blot assays also detected an upregulated protein level of PCNT in HCC tissues. In addition, higher PCNT expression at mRNA and protein levels correlated with unfavorable clinicopathologic parameters and poor prognosis. Moreover, further multivariate cox analyses even determined PCNT as an independent risk factor for OS and RFS. These results suggested that PCNT may serve as a prognostic biomarker for HCC. Based on IHC staining scores and multivariate cox regression, a predictive nomogram was constructed to accurately evaluate the 1-, 3-, and 5-year OS periods. The C-index, time-dependent ROC, and calibration curves validated the satisfactory prognostic performance of the nomogram. DCA curves exhibited the highest net benefits of the combined nomogram model. This nomogram may be a new simple tool for accurately predicting the OS of patients with HCC. Besides, our functional experiments indicated that PCNT knockdown prohibited the proliferation, migration, and invasion capacities of HCC cells, validating the pro-oncogenic effects of PCNT. Due to the use of IHC staining being more prevalent, postoperative PCNT IHC examination combined with nomogram can help predict the recurrence and prognosis of HCC patients.

It is noteworthy that almost none of studies have detected the circulating level of PCNT in cancer patients, and the expression level in plasma is more conducive to clinical application. Singh et al. detected elevated PCNT expression levels in circulating tumor cells and validated PCNT as a surrogate marker of centrosome amplification in metastatic breast cancer [32]. Thus, this finding encourages us to further detect the PCNT expression in plasma or circulating tumor cells in HCC patients, which may provide direct evidence in support of PCNT in the diagnosis and prognosis of HCC.

The immune cell infiltration and expression of immune checkpoint inhibitor-related genes in the tumor microenvironment play key roles in the initiation, development, and immunotherapy resistance of cancers [34–36]. Our results demonstrated that PCNT was associated negatively with the immune, and stromal scores and associated positively with Th2 cells, T helper cells, and Follicular helper T cells. In addition, PCNT positively correlated to a few immune checkpoint inhibitor-related genes. Interestingly, the malignant cells and immune cells detected higher PCNT levels than the stromal cells and normal hepatocytes in the single-cell RNA sequencing analysis. The results of single-cell RNA sequencing analysis found that PCNT was mainly expressed in monocytes, macrocytes, and dendritic cells, which differed from the results of the TCGA database. We hypothesized that tissue and cell origin contributed to the difference in results. On the whole, our data suggested that PCNT may serve as an essential regulator in the tumor microenvironment, as well as a promising target for immunotherapy in HCC. However, the specific mechanisms of how PCNT regulated the tumor microenvironment remain unclear and which need further in-depth analysis.

As an important cytoskeleton protein, a body of evidences has shown that PCNT was linked to the regulation of the cell cycle in cancers or non-neoplastic diseases [11, 37, 38]. Steinfeldt and colleagues reported that alternative splicing of PCNT helps to control the cardiomyocyte cell cycle arrest of newborns [39]. The PCNT mutations cause cell cycle and division abnormalities, thereby leading to microcephalic osteodysplastic primordial dwarfism type II syndrome, an autosomal recessive disease [38]. Our KEGG and GSEA analysis elucidated that PCNT was remarkably involved in cell cycle signaling. In addition, *in vitro* experiments validated that protein levels of three cell cycle-related proteins (CCNA2, CDK4, and CCND1) significantly decreased in HCC cells after knockdown PCNT expression, suggesting that PCNT might affect the progression of HCC through regulating the cell cycle signal. We acknowledge, however, some limitations. The main shortcoming of this study is that *in vitro* experiments verified the role of PCNT expression in HCC, but there is lack of *in vivo* experimental verification and requires further experimental research. Secondly, the detailed molecular mechanism and signaling pathway of cell cycle arrest induced by PCNT in HCC have not been further investigated, which was the focus of our further research. Of course, we will also promote the clinical application of PCNT expression in the diagnosis and treatment of HCC.

## CONCLUSION

In summary, our results showed that both PCNT mRNA and protein expression were upregulated in HCC tissues and correlated with unfavorable clinicopathological characteristics and poor prognosis. In addition, a predictive nomogram was constructed to accurately evaluate the 1-, 3-, and 5-year OS periods. Furthermore, PCNT expression level was correlated with mutation profiles, immune cell infiltration and immune checkpoint-related gene expression in the tumor microenvironment. Finally, the *in vitro* experiments indicate that PCNT promoted tumor progression by inhibiting cell cycle arrest.

## METHODS

### Expression of PCNT in HCC

TCGA-LIHC and GEO (GSE54236 and GSE76427 datasets) databases were employed to investigate the PCNT mRNA level between HCC tissues and adjacent normal liver tissues. Associations between PCNT expression levels and clinicopathological characteristics were investigated in TCGA-LIHC and UALCAN databases. Kaplan-Meier Plotter database was employed to explore the associations of PCNT expression and overall survival (OS) and recurrence-free survival (RFS).

### Patients and sample collections

We collected the tumor and adjacent liver tissues from 174 HCC patients who underwent radical hepatectomy at the Jiangxi Provincial People’s Hospital from 2012 to 2017. Detailed clinicopathologic parameters and follow-up information on these patients were also collected. Patients were chosen based on the following criteria: over 18 years old, without additional extra-hepatic metastases, had not received prior chemotherapy or radiotherapy, only one liver resection, only one liver resection, and pathologically confirmed as having HCC after surgery. The retrospective data acquisition was approved by the ethics committee of Jiangxi Provincial People’s Hospital (Nanchang, China).

### Immunohistochemistry assay and evaluation

174 formalin-fixed, paraffin-embedded HCC samples were sectioned, dewaxed, and dehydrated for immunohistochemical (IHC) assay. The detailed IHC procedure was performed as described earlier [40]. The primary antibody (anti-PCNT, ABIN7094416, 1:200, antibodies-online, Beijing, China) was incubated with the sections. The 3,3′-diaminobenzidine and hematoxylin were employed for section staining. IHC staining was quantitatively determined using Image J software (National Institutes of Health, Wellington, USA). The staining quantitation was based on the following scores: 0, cells stained negative; 1, ≤25% positively stained cells; 2, 26–50% positively stained cells, 3, 51–75% positively stained cells; 4, more than 76% positively stained cells. Staining score 1 or 2 was defined as low PCNT expression, and 3 or 4 was defined as high PCNT expression.

### Cell culture and shPCNT transfection

The human hepatocytes LO2 (SNL-141, China) and HCC cell lines (Huh7: SCSP-526; HepG2: HB-8065; and Hep3B: HB-8064) were obtained from American Type Culture Collection and were cultured in DMEM (Solarbio, Beijing, China) with 10% fetal bovine serum (FBS, Solarbio, Beijing, China) in a moist atmosphere at 37°C with 5% CO2. Lipofectamine^®^ 3000 (L3000001, Invitrogen, USA) was utilized for shPCNT or shRNA control (shCtrl) transfection according to the manufacturer’s protocol (Zolgene, Fuzhou, China). The shPCNT sequence was the following: shPCNT#1: 5′-CGGTCTTGTGGAACCAGAA-3′; shPCNT#2: 5′-GGACAGAACUUUGUCUGAAUG-3′; shPCNT#3: 5′-GCAGCUGAGCUGAAGGAGATT-3′.

### RT-qPCR

The RT-qPCR assay was used to investigate PCNT mRNA expression in hepatocytes and HCC cell lines. The total RNA was isolated using RNeasy kit (Qiagen, Hilden, Germany) and synthesized into cDNA by M-mlv reverse transcriptase kit (Takara Bio, Beijing, China). The procedure of reverse transcription and qRT-PCR methods were performed as described earlier. The quantitation of PCNT mRNA was detected using the 2−ΔΔCt method. The primers for PCNT were F: 5′-CCTGTCCCCAGCGAAAGAG-3′ and R: 5′-ACAGCTCCGGCGCTGGAG-3′. The primers for GAPDH were F: 5′-CAGAAGACTGTGGATGGCCC-3′ and R: 5′-AGTGTAGCCCAGGATGCCCT-3′.

### Cell viability measurement

Cell Counting Kit-8 (CCK-8) kit (Dojindo, Kumamoto, Japan) was utilized to detect the cell viability of HCC cells, which were transfected with shPCNT or shCtrl. The prepared cells were harvested and resuspended into wells of a 96-well plate and then were cultured for 0 h, 24 h, 48 h, 72 h, and 96 h. Finally, 450 nm OD values were determined by microplate reader to quantify the cell viability.

### Transwell migration and invasion assays

We determined the migration and invasion capacities of HCC cells by transwell assays with 8.0-μm pore size transwell membranes. We suspended the HCC cells (5 × 10^3^ cell/well) stably transfected with shPCNT or shCtrl in the serum-free medium and then seeded them into the up well of the 24-well plate. The bottom chamber contained fully 10% FBS. For migration assay, the chamber was coated without Matrigel. For invasion assay, the chamber was precoated with Matrigel (BD Biosciences, USA). After the cells were seeded and incubated for 24 hours at 37°C with 5% CO2, methanol and crystal violet were used to stain the cells in the bottom chamber. Finally, we randomly selected fields under the microscope to count the number of migratory or invasive cells and took pictures.

### Western blot

We performed the western blot (WB) assays to investigate the protein expression in HCC samples and transfected cells. The detailed WB procedure was performed as described earlier [40]. We used the following primary antibodies: anti-PCNT (ABIN7094416, 1:1000, antibodies-online), anti-CCNA2 (ab181591, 1:1000, Abcam), anti-CDK4 (ab108357, 1:1000, Abcam), anti-CCND1 (ab255348, 1:1000, Abcam), anti-GAPDH (ab8245, 1:1000, Abcam). The second antibody was HRP-conjugated Affinipure Goat Anti-Mouse/Rabbit IgG (SA00001-1/SA00001-2, Proteintech).

### Association of PCNT expression with gene mutation, TMB, and MSI

The somatic mutation data in patients with HCC were obtained from TCGA-LIHC datasets. The differential analysis of mutated genes in high and low PCNT subgroups was performed using “oncoplot” R package. Then, the “maftools” R package was employed to investigate the associations of PCNT expression with tumor mutation burden (TMB), microsatellite instability (MSI), Mutant-allele tumor heterogeneity (MATH), tumor purity, tumor neoantigen, tumor ploidy, homologous recombination deficiency (HDR), and loss of heterozygosity (LOH), and these data were obtained from prior publication.

### Immune microenvironment analysis

We utilized the “Estimate” R package to explore the relationships of PCNT expression with the stromal, immune, and ESTIMATE scores in HCC. Then, we employed the CIBERSORT algorithm and Mann-Whitney *U* test to investigate the difference in immune cell abundance between high and low PCNT expression patients. We next analyzed the expression level of 25 immune checkpoint inhibitor-related genes and calculated the Pearson correlations of these genes with PCNT expression.

### Single-cell RNA sequencing analysis

TISCH2, a web server that provides detailed cell-type annotation at the single-cell level, was employed to perform single-cell RNA sequencing and investigate expressed differences of PCNT mRNA in malignant cells, hepatocytes, stromal cells, and different types of immune cells [41]. LIHC_GSE146115, LIHC_GSE125449_aPDL1aCTLA4 and LIHC_GSE140228_10X datasets were selected to determine correlation of PCNT with tumor microenvironment.

### Establishment and validation of prognostic nomogram

A prognostic nomogram containing all independent prognostic factors identified from a cohort with 174 HCC patients was established for clinical use. In addition, time-dependent ROCs, concordance indexes (C-index), and calibration curves were employed to assess the effectiveness of the nomogram. The decision curves analysis (DCA) was used for clinical decisions.

### Co-expression gene analysis of PCNT

We identified the co-expressed genes of PCNT in the LinkedOmics web server (http://linkedomics.org/admin.php) by using RNAseq data in TCGA_LIHC datasets. The genes with Pearson’s value greater than 0.5 and with *P* < 0.05 were identified as co-expressed genes of PCNT. We then investigated the interactions between these co-expressed genes with PCNT and the protein-protein interaction (PPI) network was constructed and visualized. The prognostic value of these co-expressed genes of PCNT was also investigated.

### Functional enrichment analyses

We performed gene ontology (GO) analyses with the “clusterProfiler” R package on co-expressed genes to explore the biological process, cellular component, and molecular function of PCNT. Kyoto Encyclopedia of Genes and Genomes (KEGG) pathways that PCNT related were identified in the LinkedOmics database. We next divided HCC patients in TCGA_LIHC datasets into high and low PCNT expression subgroups based on median PCNT expression value. The gene set enrichment analysis (GSEA) was performed to uncover the potential carcinogenesis signaling pathways of PCNT involved in HCC.

### Statistical analysis

The statistical analyses were conducted by SPSS 23.0 software. The figures were drawn using GraphPad Prism 8.0 and R (version 4.1.0) software. The correlations between PCNT expression with clinicopathological parameters were analyzed using the Pearson chi-square test. Between-group comparisons were performed using a two-tailed Student’s *t*-test and Mann–Whitney rank sum test. Survival rates were compared using a K-M plotter with log-rank tests. The prognostic factors were identified using the Cox hazard regression model. *P* < 0.05 represented statistically significant.

### Availability of data and materials

The datasets generated for this study can be found in the GEO database (https://www.ncbi.nlm.nih.gov/geo/) and TCGA database (https://portal.gdc.cancer.gov).
